# Biomaterials in Neurodegenerative Disorders: A Promising Therapeutic Approach

**DOI:** 10.3390/ijms21093243

**Published:** 2020-05-04

**Authors:** Matteo Bordoni, Eveljn Scarian, Federica Rey, Stella Gagliardi, Stephana Carelli, Orietta Pansarasa, Cristina Cereda

**Affiliations:** 1 Dipartimento di Scienze Farmacologiche e Biomolecolari (DiSFeB), Centro di Eccellenza sulle Malattie Neurodegenerative, Università degli Studi di Milano, Via Balzaretti 9, 20133 Milano, Italy; matteo.bordoni@mondino.it; 2 Department of Brain and Behavioural Sciences, University of Pavia, Via Forlanini 6, 27100 Pavia, Italy; eveljn.scarian@mondino.it; 3 Genomic and post-Genomic Center, IRCCS Mondino Foundation, Via Mondino 2, 27100 Pavia, Italy; stella.gagliardi@mondino.it (S.G.); cristina.cereda@mondino.it (C.C.); 4 Department of Biomedical and Clinical Sciences “L. Sacco”, University of Milan, Via Grassi 74, 20157 Milan, Italy; federica.rey@unimi.it (F.R.); stephana.carelli@unimi.it (S.C.); 5 Pediatric Clinical Research Center Fondazione “Romeo ed Enrica Invernizzi”, University of Milan, Via Grassi, 74, 20157 Milan, Italy

**Keywords:** biomaterials, neurodegenerative disorders, spinal cord injury, regenerative medicine, tissue engineering, stem cells

## Abstract

Neurodegenerative disorders (i.e., Alzheimer’s disease, Parkinson’s disease, amyotrophic lateral sclerosis, and spinal cord injury) represent a great problem worldwide and are becoming prevalent because of the increasing average age of the population. Despite many studies having focused on their etiopathology, the exact cause of these diseases is still unknown and until now, there are only symptomatic treatments. Biomaterials have become important not only for the study of disease pathogenesis, but also for their application in regenerative medicine. The great advantages provided by biomaterials are their ability to mimic the environment of the extracellular matrix and to allow the growth of different types of cells. Biomaterials can be used as supporting material for cell proliferation to be transplanted and as vectors to deliver many active molecules for the treatments of neurodegenerative disorders. In this review, we aim to report the potentiality of biomaterials (i.e., hydrogels, nanoparticles, self-assembling peptides, nanofibers and carbon-based nanomaterials) by analyzing their use in the regeneration of neural and glial cells their role in axon outgrowth. Although further studies are needed for their use in humans, the promising results obtained by several groups leads us to suppose that biomaterials represent a potential therapeutic approach for the treatments of neurodegenerative disorders.

## 1. Introduction

### 1.1. Neurodegenerative Disorders

Neurodegenerative disorders (NDs) are a broad class of disorders characterized by the progressive loss of disease-specific neural population and they include Alzheimer’s disease (AD), Parkinson’s disease (PD), amyotrophic lateral sclerosis (ALS), and spinal cord injury (SCI). NDs are a growing cause of mortality worldwide, particularly in the elderly population. There are many NDs such as amyloidosis, taupathies, α-synucleinopathies, and TDP-43 proteinopathies, which are heterogeneous in clinical presentations and underlying physiology, although often presenting with overlapping features [1]. The majority of NDs can be divided into familial and sporadic form, depending on the familial history of the disease. Moreover, NDs are often due to misfolding of physiological proteins in both neural and glial cells [2].

NDs are usually considered as separate clinical entities because they target different brain areas and have distinct symptoms. However, they share several common features at the genetic, molecular, or cellular level (e.g., early vascular dysfunction, the aggregation and spread of misfolded proteins, selective vulnerability of particular neurons, and activation of immune responses) [3]. Sweeney and colleagues investigated the role of cerebral blood flow and blood–brain barrier (BBB) integrity in the pathogenesis of several NDs, finding many similarities [4]. Another hallmark of NDs is the accumulation of misfolded proteins. Although the proteins are often different for each ND, the process of protein misfolding and aggregation is remarkably similar, leading to cellular dysfunction, loss of synapses, and finally brain damage. Furthermore, such proteins can usually self-propagate through seeding, spreading the pathological abnormalities between cells and tissues [5]. Altered cellular mechanisms are also very similar between NDs, and they include mitochondrial defects, dysfunctions in autophagic–lysosomal pathways, synaptic toxicity, and more emerging mechanisms such as the roles of stress granule pathways and liquid-phase transitions [6]. Finally, for each ND, microglial cells have been reported to be implicated in neurodegeneration, in particular, because the microglial responses can shift from neuroprotective to a deleterious role [7].

#### 1.1.1. Alzheimer’s Disease

AD is the most common ND and the first cause of dementia. There are nowadays about 46.8 million people suffering from AD worldwide. Moreover, the number of AD patients is expected to double nearly every 20 years [8,9]. In most patients, the causes of AD are idiopathic. It has been proven that genetics is responsible for familial forms of this disorder, which constitute 1% of all cases. Early onset AD has been linked to mutations in four genes: the genes encoding amyloid precursor protein (APP), presenilin 1, presenilin 2, and tau protein [10].

The classical symptoms of AD include dementia, memory and spatial awareness impairment, movement dysfunction, depression, delusion, and hallucination. Patients also experience anomic aphasia, acalculia, and apathy [10,11].

Many phenomena leading to AD remain unclear, although there has been significant progress in the research explaining AD-related changes. The most widely accepted hypothesis is the so-called amyloid cascade. It consists in the accumulation of amyloid beta (Aβ) due to genetic defects (i.e., APP, presenilin 1, presenilin 2), environmental factors, and other stressors. These senile plaques induce an immune response, leading to inflammation, tau hyperphosphorylation, and its aggregation in tangles [12] that lead to neuron degeneration and death, and impairing neurotransmission in many brain regions [13].

There are two main symptomatic treatments for AD: acetylcholinesterase inhibitors (i.e., donepezil, galantamine, and rivastigmine) that increase the availability of acetylcholine at synapses, and the noncompetitive *N*-methyl-d-aspartate (NMDA) receptor antagonist memantine, which modulates the opening of the calcium channel [14].

#### 1.1.2. Parkinson’s Disease

PD is the most common movement disorder and the second most common ND. The incidence of PD is eight to 18 per 100,000 person-years overall, and prevalence reaches 1% in subjects over 60 years and 3% over 80 years with a higher incidence in men than in women [15]. As in other NDs, there are two forms: the sporadic form, which represents 90% of the cases, with an early onset, and the familial form, which represents 10–15% of the cases with an onset beyond 50 years [16,17].

PD is characterized by the degeneration of dopaminergic neurons of the substantia nigra *pars compacta*, which projects to the basal ganglia and to the striatum [18]. It causes motor symptoms such as the classical triad: tremor, bradykinesia, and rigidity.

In PD, α-synuclein, a protein present in dopaminergic terminals important for synaptic vesicles trafficking, misfolds, forming oligomers that aggregate in the so-called Lewy bodies [19]. There are many theories about PD causes and, although different, all of them involve α-synuclein aggregation [20]. Some theories suggest that PD is caused by mitochondrial dysfunction [21], oxidative stress, high levels of dopamine, and microglia impairment [22,23]. Like a vicious circle, all these theories can explain α-synuclein aggregation and α-synuclein aggregation can cause all these effects.

The only therapies available for PD are symptomatic ones and the most common are the treatments with L-DOPA or with dopamine agonists.

#### 1.1.3. Amyotrophic Lateral Sclerosis

ALS, also called “Lou Gehrig’s disease,” is a common ND with an incidence of 2–3 per 100,000 individuals in Europe and 0.7–0.8 per 100,000 individuals in Asia, with a survival of 2–5 years after onset, which occurs around 65 years of age [24].

It affects both upper and lower motor neurons in the cortex, brainstem, and spinal cord, and it is associated with weakness, muscle atrophy, and spasticity [24,25].

Although 90–95% of the cases are sporadic, the familial form may represent 5–10% of the total number of patients, with many genes involved. The most common mutations in both familial and sporadic cases involve SOD1, TDP-43, FUS, and C9orf72, with the expansion of the GGGGCC hexanucleotide. However, in recent years, thanks to massive parallel sequencing (whole-genome sequencing and whole-exome sequencing), progress has been made in the identification of new genes involved in rare variants [26]. Although the causes of ALS differ, a common histopathological sign is the presence of intracytoplasmic inclusions such as Bunina bodies, but also SOD1, ubiquitylated, and TDP-43 inclusions [27].

To this day there are only two drugs approved, the glutamate antagonist Riluzole and the antioxidant Edaravone, but they have a mild effect.

### 1.2. Spinal Cord Injury

SCI is a devastating neurological condition that leads to loss of sensory and motor functions. It can also lead to other significant problems such as bladder and bowel dysfunction, infections, chronic pain, and cardiac and respiratory issues [28]. SCI impacts on the lives of many people worldwide, with an estimated number of 27 million people living with this condition [29].

SCI occurs as a result of a traumatic event (i.e., motor vehicle accident, fall, sporting injury or an act of violence). The trauma is followed by a multifactorial process that involves a complex series of molecular and cellular events composed of two different phases. The first step is represented by local cellular damage caused specifically by the traumatic injury and is followed by many secondary reactive processes including ischemia, inflammation, edema, cell death, axonal degeneration, gliosis, and formation of scar tissue [30,31,32].

Treatments currently available for SCI are limited and mainly focus on adaptive and rehabilitative therapies as well as the management of secondary complications [30,33].

### 1.3. Current Treatments

Although many studies have focused on the causes of NDs and on possible therapies, the treatments currently available are limited and they only address symptoms and not the mechanisms underlying the pathology.

Many problems arise in the treatment of these pathologies. One main problem is the permeability of the BBB, which prevents administered drugs from reaching therapeutic levels in the brain. For this reason, there is the need to develop bioactive compounds able to cross the BBB [34]. Other hurdles in the treatment of these diseases include the need for such treatments to be safe for a long period. Finally, there is the need to identify the persons affected by NDs in early stages, as many of them do not present symptoms at the beginning of the illness [35,36].

A promising strategy to improve intracerebral drug delivery is represented by gene therapy, which, by altering or inducing the expression of specific genes using modified viral vectors derived especially from adeno-associated viruses (AAV), can have a great role in neuroprotection, restoration, and correction of the underlying pathogenic mechanisms. For example, SOD1 is known to play an important role in the progression of ALS [37] and an injection of AAV carrying a SOD1 silencing RNA in mice expressing a mutant SOD1 produces long-term suppression of MNs disease [38]. Moreover, the development of a convection-enhanced delivery (CED) in the 1990s and of a real-time magnetic resonance imaging (MRI)-guided CED in recent years, has allowed for monitoring of the infusion of the drug via co-administration of an MRI-contrast agent to have a specified therapy in the anatomical regions of interest [34]. Many clinical trials, especially for PD [39,40,41] and AD [42], have begun using this technique.

In recent years, many studies have focused on protein misfolding. They aim to inhibit the production of the protein involved in the disease, inhibit the aggregation of such proteins, remove and prevent their spreading, and to ease their toxic effect. For these purposes, chemical agents were developed that were able to inhibit aberrant protein aggregation, chemical modulators of autophagy, and specific antibodies in order to eliminate any misfolded proteins [27,43]. Furthermore, many studies have focused on the modulation of chaperone proteins and proteasomal components [27].

Another therapeutic angle that has emerged in the past few years is the use of stem cells as a new approach in the treatment of these pathologies. Stem cells have the ability to renew themselves continuously and to differentiate into almost all cell types. Different stem cell lines have been explored in recent years [44]. Mesenchymal stem cells (MSCs) appear to be the most suitable because of their high availability, satisfactory amounts, and very low immunogenicity. Moreover, they can also differentiate into neurons and glial cells when cultured under specific conditions [45]. MSCs can be obtained from several tissues, but the most studied MSCs are those from the umbilical cord (UC-MSCs), bone marrow (BMSCs), and adipose tissue (ASCs) [46].

Despite having been tested in many studies and clinical trials for different NDs [47], MSCs have many limitations that need to be overcome for their clinical therapeutic use. Indeed, the complexity of the neural tissue precludes the potential of many therapeutic approaches based on stem cells. The most important factor is the lack of correlation between the in vitro and in vivo behavior of stem cells, as the microenvironment is essential for their differentiation [48]. For this reason, an important challenge to overcome for the therapeutic use of stem cells in vivo is represented by the understanding of the surrounding chemical and physical signals as well as cell–cell interactions [49]. Such features can be easily studied by introducing biomaterials in both in vitro and in vivo models. Biomaterial scaffolds hold great promise for the generation of innovative 3D culture systems and for in vivo applications aimed at improving the effect of stem cells in the treatment of brain injuries [50]. Indeed, the ability of bioengineered scaffolds to mimic the environment of the extracellular matrix (ECM) allows for better cellular infiltration, resulting in improved proliferation and correct differentiation. Finally, the ability to control cellular behavior through functionalization suggests that these scaffolds are ideal to be combined with cells in the field of neural regeneration [50].

## 2. Biomaterials

### 2.1. Characteristics of Biomaterials

The science and engineering of biomaterials is a new emerging field. In the past, biomaterials were defined as “non-vital materials used in medical devices, intended to interact with biological systems” and they were considered only in the field of medical devices [51,52]. Nowadays, the definition is more general, and a biomaterial is considered as “any material, except to drugs, that interacts with living tissues and performs a particular function without causing adverse effects” [52,53].

Biomaterial evaluation is based on their safety and performance. The biomaterial should be biocompatible, biofunctional, bioinert, biodegradable, and sterilizable to avoid irritation and rejection [52,54,55]. The biomaterial, its degradation products, and its sterilization residuals, should not cause adverse effects in the host tissue (biocompatibility). For this purpose, there are many tests that can be used to evaluate the toxicity, pyrogenicity, inflammation potential, effects after implantation, hemocompatibility, and sensitization potential [52,55,56]. Every biomaterial should also be tested for the clinical effectiveness, that is, if it can produce the desired effects (biofunctionality). A bioinert material is a material that has no chemical reaction with the tissue. Finally, a biodegradable material is a material that solubilizes, degrades, or is adsorbed after a certain period of contact with the tissue.

Moreover, the choice of the correct biomaterial is based on physical (wettability, filler, roughness, softness, and chemical composition), chemical (corrosion properties and surface functional groups), mechanical (ductility, tensile strength, yield strength, compression strength and fatigue), and biological properties [55].

Biomaterials are important tools not only in the field of medical devices and regeneration, but also for the study of different types of cells, involved in different pathologies, in a more physiological environment. Many are the biomaterials studied and developed in the last years, but the ones that have shown the best characteristics are hydrogels, nanofibers, carbon-based nanomaterials, and cell-free scaffolds [50] as reported in the Table S1 (supplementary material).

### 2.2. Hydrogels

Hydrogels are three-dimensional, highly hydrated, water-insoluble polymer networks held together by chemical and/or physical crosslinks. They are called physical gels if the molecular entanglements or secondary forces are the most important actors in the formation of the network; these types of gels are reversible when changing conditions such as temperature or pH. In chemical gels, the network is obtained by cross-linking the polymers; these second types of gels can be charged or uncharged. There are two methods to prepare chemical hydrogels, and these are the three-dimensional polymerization in which a hydrophilic monomer is polymerized by a cross-linking agent and then purified, and the direct cross-linking of water-soluble polymers, which do not need purification procedures [57,58]. The high content of water, the porosity, and the soft consistency of the hydrogels allow them to transport oxygen, nutrients, and soluble factors to simulate living tissues better than other biomaterials.

Many types of hydrogels have been studied and used, alone or in a mixture at different ratios in the field of neurodegeneration and regeneration. These are natural hydrogels like hyaluronic acid (HA), xyloglucan, collagen, alginate sodium, and gelatin, but also synthetic ones such as polyacrylamide and polyethylene glycol.

Hydrogels may be used for drug and cell delivery in the study and treatment of NDs. Different types of cells can be encapsulated in 3D-hydrogel structures, which will then be implanted in the brain tissue, or the hydrogel itself can be implanted in a pregel state, which will then form a gel directly in the brain, allowing a more precise localization of the implanted cells. The 3D structures allow the growth of cells in a permissive environment in which there are both the entry of oxygen and nutrients and the output of waste products. Moreover, the 3D structure can act as a barrier for the inflammatory cells or adverse factors in the host tissue [59].

*AD.* In AD, the studies focused mainly on drug delivery. Peptide-amphiphile hydrogels have been studied for the release of the antioxidant and neuroprotective compound curcumin [60] and hydrogels made of gellan gum and xanthan gum for the release of Resveratrol [61]. The encapsulation of VEGF-secreting fibroblasts into alginate reduced amyloid-β deposition in APP/PS1 mouse models. Moreover, many studies have shown that the encapsulation of neural stem cells (NSCs) in different types of hydrogels improved stem cell survival and the cognitive capacities in mouse models [62,63].

*PD.* With regard to PD, many authors have focused on dopamine delivery. Senthilkumar and co-authors in 2007 and, more recently, Ren et al. in 2017, studied the effect of dopamine delivery from dextran/gelatin and chitosan/ gelatin hydrogels, respectively. Both types of hydrogel showed good release of the drug and Senthilkumar et al. noticed a behavioral and motor improvement in PD mice after the treatment [64,65]. Moreover, several neurotrophic factors such as BDNF and GDNF and epidermal growth factor have been delivered and also combined with embryonic stem cell-derived dopaminergic neurons in different types of hydrogels. In 2019, Ucar and Humpel encapsulated GDNF in collagen-hydrogels and noticed an enhancement of dopaminergic cell survival [66], whereas in 2016, Wang and colleagues had already noticed that GDNF released by a poly-L-lactic acid/xyloglucan hydrogel supported nerve fiber outgrowth and reinnervation of the striatum in a mouse model of PD [67]. Even more interesting is the possibility of implanting constructs that mimic a neural pathway. In 2018, Struzyna and colleagues encapsulated embryonic stem cell-derived dopaminergic neurons in hydrogel micro-columns to reconstruct axonal tracts of the nigrostriatal pathway [68].

*ALS.* In contrast, few authors have worked on the use of hydrogel in ALS. Osaki and colleagues developed a 3D human motor unit model in a collagen/Matrigel microfluidic device. They co-cultured MN spheroids and 3D muscle fiber bundles to mimic the pathological conditions of motor units of patients with ALS [69]. In 2019, Fantini and colleagues studied the effect of a hydrogel composed of 6% sodium alginate and 4% gelatin on the viability of different types of cells including induced pluripotent stem cells (iPSCs) and NSCs. Viability was maintained and the hydrogel printed in a 3D structure allowed for the 3D organization of the cells, mimicking the environment of the tissue. These results open the possibility of a new model for the study of ALS, especially in the neuromuscular plaque [70].

*SCI.* With regard to SCI, hydrogels can be used as scaffolds to fill the lesion cavity and re-connect the two nerve ends. Stem cells and other biomolecules can be encapsulated in hydrogels, allowing regeneration and plasticity. Studies have shown that when neural stem cell progenitors (NSPCs) mixed in platelet-derived growth factor-A encapsulated in a hyaluronan-methylcellulose gel were transplanted into a rat SCI model, their differentiation and the differentiation of oligodendrocytes was enhanced [71]. Fibrin-based hydrogels have been utilized to deliver stem cells and growth factors in SCI rat models. It was demonstrated that embryonic neural stem/progenitor cells (ENSPCs) encapsulated in this type of hydrogel with different growth factors increased in number, which also happens for ESNPC derived NeuN+ mature neurons [72]. Pertici and colleagues demonstrated that poly(*N*-[2-hydroxypropyl]-methacrylamide) hydrogel implantation into a rat model of SCI induced locomotor and neurophysiological improvements. This is probably because the hydrogel provides a more suitable environment for regenerating axons and prevents secondary injury and glial scar formation, resulting in higher neuroplasticity [73]. A 3D-bioprinted hydrogel scaffold tailored to the dimensions of the rodent spinal cord was demonstrated to regenerate axons, restoring synaptic transmission and improving functional outcomes [74]. A recent study also demonstrated that hydrogels could eliminate the cystic cavities that form after SCI and which represent the major obstacle for tissue repair. An imidazole-poly(organophosphazenes) (I-5) hydrogel can, in fact, interact with the ECM and with macrophages of the host tissue, and inhibit the formation of these cavities [75].

### 2.3. Nanoparticles

As already mentioned, the BBB interferes with the passage of drugs to the brain. Larger systemic doses of drugs are required to achieve the therapeutic levels in the brain, with adverse effects in the body. For this reason, there is a necessity for strategies that are able to enhance their concentration in the brain [34].

Nanotechnology, which consists of the use of materials or devices on a nanometric scale (1–100 nm), has emerged in the last years as a promising approach to treat different pathologies, like neurodegenerative disorders. There are different types of nanotechnology devices such as nanoparticles, nanofibers, nanotubes, nanospheres, and nanogels [76].

Nanoparticles (NPs) are one of the most preferred nanostructures because of their characteristics: small size, vast surface, and surface–volume ratios. They can be composed of different materials such as ceramics, metals, oxides, salts, and polymers. Silica NPs, especially the mesoporous silica NPs with a pore size of 2–50 nm, are the most frequently used due their large surface area and pore volume; furthermore, it is easy to control their size and they have a good biocompatibility.

Even so, there are many other types of NPs used in drug delivery: polymer NPs, which are easier to synthesize and less expensive, gold NPs, protein NPs, and liposomes, which are lipid NPs [76].

*AD.* The NPs used in NDs began years ago. With regard to AD, many authors have focused on the delivery of common and available drugs through NPs. Acetylcholinesterase inhibitors (e.g., Rivastigmine, and anti-amyloid and anti-transferrin receptors) have been loaded in different types of NPs, demonstrating an increased delivery in rat brains and positive therapeutic outcomes in amnesic mice [77,78]. The same happens when loading curcumin in this type of NP [79]. NPs have also been used in metal chelation therapy, which uses metal-chelating compounds to reduce cellular oxidative stress in AD. It was demonstrated that the use of NPs loaded with chelating compounds improved drug delivery with benefits both in vitro and in vivo [80]. Moreover, the encapsulation of epigallotechin-3-gallate, a natural α-secretase promoter, and antioxidant compounds in lipidic NPs, enhances its neuronal availability in vivo [81]. More interestingly, lipidic nanoparticles were studied as vaccine carriers against aggregated Aβ peptides and against pathological conformers of phosphorylated tau proteins, with successful results [82].

*PD.* Levodopa is the most frequently used drug in the treatment of PD, although it causes many adverse effects. It was demonstrated that the loading of Levodopa methyl ester in poly-(lactic-co-glycolic acid) (PLGA) NPs abolishes adverse effects of Levodopa such as dyskinesia [83]. Moreover, it was shown that the use of lipidic NPs improved dopamine delivery in the brain [84]. Dopamine agonists (e.g., Bromocriptine, and NADPH oxidase inhibitors) were also encapsulated in NPs to improve their delivery in the brain with benefits on PD symptoms [85,86].

*ALS.* With regard to ALS, the use of nanoparticles recapitulates all the strategies adopted for the other NDs: lipidic nanoparticles [87], the use of curcumin-loaded NPs [88], the use of gold NPs [89], and of course, the use of NPs for the delivery of available drugs such as Riluzole [90]. One of the main characteristics of ALS is the high level of oxidative stress. For this reason, oxidative stress is a target in the treatment with cerium oxide NPs (CeO2 NPs), which have regenerative antioxidant properties [91,92]. DeCoteau and colleagues demonstrated that CeO2 NPs enhanced strength and prolonged the life-span of SOD1G93A mice [93].

*SCI.* In SCI, the use of NPs has shown many advantages in drug delivery: drug-loaded NPs can give better pharmacokinetics and biodistribution than the free drugs [94]. Methylprednisolone, a corticosteroid medication, is already used to treat SCI. Many groups have demonstrated that the use of this treatment in combination with NPs has a better outcome than with methylprednisolone alone in vivo, with a reduction of the lesion and locomotor improvements [95,96]. Moreover, the use of poly-ε-caprolactone-based NPs in combination with minocycline, an anti-inflammatory drug, allows for the reduction of inflammatory response acting on resident microglia cells [97]. However, in the last few years, new methods have emerged to reduce inflammation in SCI. Park and colleagues reprogrammed circulating monocytes and neutrophils through PLGA nanoparticles. The authors noticed that the treatment caused a downregulated expression of proinflammatory factors and an enhanced expression of anti-inflammatory and pro-regenerative genes, leading to an increased number of regenerating axons, increased myelination, and enhanced locomotor function [98]. In SCI, magnetic nanoparticles were used to track specific cells after implantation [99]. It was observed that the injection of stem cells with magnetic nanoparticles enhanced transfection efficiency and cell viability [100]. More recently, it was shown that the use of iron oxide nanoparticles along with electromagnetic fields induced sprouting from mature neurons and axons, significantly less demyelination and more myelinated fibers in injured rats [101]. Other factors such as neurotrophins [102,103] and the enzyme chABC [104], which has already been seen as a promising treatment for SCI, have been loaded in NPs, showing that they can induce axon regrowth with a controlled and sustained release at the site of the lesion.

### 2.4. Self-Assembling Peptides

Self-assembling peptides (SAPs) can be monomers or amino acid sequences that assemble to form nanostructures like tubes, rods, and sheets, allowing the presentation of the peptides’ chemical functionality on the surface of these structures. These assemblies have different physicochemical and biochemical activities depending on the morphology, size, and accessibility of the active surface area [105]. The self-assembly of peptides can be controlled by pH, ionic strength, temperature, or enzymatic triggers. Moreover, when multiple assemblies are put together, a supramolecular network can be established. The advantages of these structures are their biocompatibility and the ease of synthesis [50,106].

There are different types of self-assembling peptides. The simplest building blocks of these structures are dipeptides, which can assemble into ordered structures in the nanoscale order.

The surfactant-like peptides are, on the other hand, characterized by an amphiphilic structure, with a hydrophobic tail and a hydrophilic head. When dissolved in water, these types of peptides assemble to minimize the contact of the tail with the water. In this way, they can form nanotubes or nanovesicles and they can acquire a function similar to that of lipid micelles present on the lipid layer of the cells [105,107,108]. The most common modification of peptides with a hydrophobic tail and a hydrophilic head is the link with a hydrophobic alkyl chain; when combined with an alkyl chain in a water solution, the hydrophobic tail forms a 3D structure, similar to what happens during protein folding. These modified peptides form structures such as micelles, vesicles, nanofibers, and nanotubes. Moreover, modification of surfactant-like peptides produces bola amphiphilic peptides, which have two hydrophilic heads instead of one. The heads can be of two different types, forming an asymmetric bola amphiphilic peptide [105].

The ionic-complementary peptides are characterized by an alternating arrangement of negatively and positively charged residues, whereas cyclic peptides are composed of amino acids forming a cylindrical structure. Cyclic peptides allow for more stable structures than the ones composed of linear peptides.

Self-assembling peptides allow the formation of different nanostructures: nanofibers; nanotubes, which are similar to nanofibers, but with a hole in the inner side where several drugs can be loaded; nanoparticles, which range from nanospheres to various solid structures; nanotapes; and hydrogels [105].

In the last years, self-assembling peptides have been used in many fields (e.g., in drug delivery and in neuronal regeneration) with regard to NDs.

The Tat (YGRKKRRQRRR) peptide is able to penetrate through the cellular membrane, while its modified form, the Tat-polyethylene glycol-b-cholesterol (Tat-PEG-b-col) peptide, which is able to form micelles, was used for drug delivery through BBB.

*AD.* Yu et al. used Lactoferrin-modified polymersomes to deliver the neuroprotective peptide humanin in an AD mouse model, showing a better drug delivery due to these types of peptides [109]. More recently, self-assembling β-sheet peptides were being studied as a nanoplatform for vaccination. These assemblies are stable, resistant to proteolysis, and they can carry many antigenic determinants to elicit a stronger immune response [110]. To allow the growth and differentiation of stem cells in biomaterials, it is important to create an environment similar to the ECM. Along with other peptides that have been studied for the development of an ECM-like biomaterial, amyloids seem to be promising peptides for the control of stem cell behavior. In fact, their formation from soluble proteins is associated with numerous degenerative diseases. Jacob and colleagues developed amyloid nanofibrils, composed of Fmoc-protected peptides derived from β-sheet prone C-terminal Aβ42, to create a hydrogel for the development of stem cells. This hydrogel allows for the proliferation and attachment of the cells, and supports the differentiation of MSCs [111]. It was also demonstrated that the β-amyloid peptide (Aβ) influences neurogenesis [112,113]. Mehrban and colleagues demonstrated that self-assembling fibers (SAF) based on α-helical coiled-coil peptides were able to create an environment that allows not only for the attachment of NSCs, but also their differentiation in neurons [114]. The combination of stem cells with SAFs could allow the culture of cells in an in vitro environment morphologically similar to that found in vivo. The possibility of obtaining tissue specific SAFs due to specific peptide sequences or stiffness, allows for the differentiation of cells into a specific lineage. It is important not only for cell transplantation in neurodegeneration, but also for a good disease modeling [50].

*SCI.* The most predominant use of these structures has been in the study of SCI and nerve regeneration. In 2009, it was demonstrated that self-assembling peptide scaffolds composed of RAD16 allowed for reconnection of the injured spinal cord and axonal regeneration, improving locomotor functional recovery in injured rats [115]. RAD16-I and RAD16-II, commercially available SAPs that assemble due to complimentary charge and hydrophobic interactions, have been proved to be a good substrate for neurite outgrowth [116]. SKPPGTSS, -PFSSTKT-, and RGD motifs combined with RAD16-I increased levels of nestin, β-tubulin, and other neuronal markers [117,118]. Zhao and colleagues demonstrated that the combination of the self-assembling peptide QL6 with neural precursor cells (NPCs) enhanced neuro-behavioral recovery, increased neuronal conduction, and improved survival [119]. In the same year, it was demonstrated that QL6 enhanced not only neuronal differentiation and axonal regeneration, but also suppressed astrocytic development with a reduction in post-traumatic apoptosis, inflammation, and astrogliosis [120]. Both these results were then confirmed by Zweckberger and colleagues in clip-compression SCI rats [121]. Recently, the ability of these structures was demonstrated to release important trophic factors. Injured rats were injected with a BDNF-loaded IKVAV peptide amphiphile hydrogel, resulting in axon preservation and astrogliosis reduction without any inflammation reaction [122]. It the importance of vascularization in these types of models has also emerged. It was seen that after the normal inflammation caused by SCI, the density of vasculature significantly decreased. For these reasons, vasculature transplantation with a blood-spinal cord barrier (BSCB), which protects from inflammation, can potentially stabilize the vasculature within the cord. With this purpose, Tran and colleagues used a self-assembling peptide scaffold RADA16-I containing microvascular cells in injured rats and noticed reduced inflammation and glial scar formation, with an increase in the density of growing axons [123].

### 2.5. Nanofibers

As previously reported, for tissue engineering, it is fundamental that scaffolds mimic the native tissue. In the brain, an axon can be considered as a bundle of ultra-small fibers with supporting cells wrapped around them. Moreover, collagen fibers and capillaries have a fascicle-like structure, important not only for neuronal survival and function, but also for contact guidance in signal transmission [124]. To mimic the fibrous structure of the brain tissue, electrospinning is used to produce nanofibers of various polymeric materials [125,126]. In particular, electrospinning has been intensively applied because of its simplicity, reproducibility, and diversity in producing fibers with various diameters and with different topographical features [127]. Another advantage of using electrospun nanofibers is the large surface-area/volume ratio, making them an optimal bioactive matrix for cell attachment, molecule loading, and functionalization in order to provide enriched biochemical and biophysical features to improve tissue regeneration. Finally, nanofibrous scaffolds incorporated with various native proteins have demonstrated a critical role for topographical cues in the functions of MSCs and their differentiation into neural cells [125].

Although many studies have been conducted for neurite regeneration and synapses formation, only a few of them have reported on the treatment of NDs, and all of them were focused on the differentiation of stem cells into dopaminergic neurons for the future treatment of PD [128,129]. Many materials can be used to produce nanofibers for neurite regeneration, leading to the obtainment of several scaffolds with various interesting features. A fundamental characteristic that nanofibers must have is the correct alignment, making them particularly useful for axon regrowth. Subramanian and colleagues tested the effect of both aligned and random nanofibers on Schwann cells and proliferation, finding an improved effect of aligned nanofibers, probably because they can mimic the fibrin cable architecture [130]. Another similar study demonstrated the effect of aligned nanofibers in iPSC-derived NSCs. Lin and colleagues found that poly-L-lactic acid nanofibers were able to promote the adhesion, growth, survival, and proliferation of NSCs, but more importantly, aligned nanofibers greatly directed neurite outgrowth from the NSCs and significantly promoted neurite growth along the nanofibrous alignment [131]. Nanofibers can be composed of synthetic materials, but they can be generated starting from natural compounds. Yin et al. tested and evaluated the effect of collagen nanofibers on a culture of NSC-derived neurons in the formation of neural networks. In particular, they investigated the miniature excitatory postsynaptic currents, finding an increased frequency in the differentiated neurons cultured on collagen nanofibers with respect to that of the collagen-coated control, suggesting the role of the topography for the beneficial effect of biomaterials [132]. Nanofibers can be loaded with drugs, and to this end, Lau and colleagues investigated the effect of chitosan nanofibers loaded with genipin. They found an increased stiffness, resistance to swelling, and lysozymal degradation of nanofibers, resulting in a better alignment and proliferation of the Schwann cell culture. Finally, in a model of peripheral nerve regeneration, neurite growth rate upon genipin-treated nanofibers demonstrated a 100% increase [133].

Nanofibers can be produced using materials with a high biocompatibility, and for this reason, many studies have been conducted in vivo, often obtaining promising results. A polycaprolactone/carbon nanofiber sheets composite was first tested in vitro and then in vivo. Farzamfar and colleagues found that such nanofiber sheets implanted in rat sciatic nerve promoted cell attachment, proliferation, and neurite out-growth [134]. Another useful functionalization of polycaprolactone has been obtained with laminin. Chang and colleagues found that the functionalization was essential for the correct organization of neural cells in a rodent model of nerve injury. Moreover, they found numerous new blood capillary-like structures around the regenerated nerve, leading the authors to hypothesize that new blood vessel formation could be one of the key factors for successful nerve regeneration [135]. Nanofibers can be loaded not only with peptides or drugs, but also with a combination between these two compounds in order to obtain a synergic effect in neural regeneration. Satish and Korrapati loaded polyvinyl cinnamate nanofibers with laminin-derived cell-adhesion peptides to improve selective neural adhesion and regeneration. Furthermore, they encapsulated triiodothyronine within the nanofibers for its sustained release to bolster regeneration and reinstate the lost functionality to the damaged nerve. They found that such nanofibers were biocompatible, improved the cell adhesion rate, and illustrated favorable interaction with cells in an adult zebrafish model. They concluded that the combination of aligned nanofibers providing topographical cues, peptides, and triiodothyronine has robust potential in restoring functionality of the injured nerve [136]. The possibility to inject nanofibers can open new possibilities for SCI. Li and colleagues developed an injectable nanofiber–hydrogel composite and implanted it in an adult rat model of spinal cord contusion. After 28 days, they found that the treated rats had a higher M2/M1 macrophage ratio, blood vessel density, neuron presence, and axon density, suggesting the fundamental role of this biomaterial in the treatment of SCI [137]. Another interesting approach for nerve regeneration is to create nerve guidance conduits. The coating of such conduits with nanofibers allows for improvement in the efficacy of regeneration. Shah and colleagues created a nerve guidance conduit cross-sectional surface composed of spiral structures and multi-channels, coupled with inner longitudinally aligned nanofibers and protein. After four weeks from implantation in a rat model with a sciatic nerve injury, they found a shorter recovery time when compared to the autograft, leading to suppose that such an approach represents a promising tool for axonal regrowth [138].

### 2.6. Carbon-Based Nanomaterials

Neural tissue and neural cells have the unique property to generate and transmit electrical stimuli. Indeed, voltage channels can be modulated by electrical stimulation, influencing not only the firing of neurons, but also cell proliferation, migration, and function [139]. For this reason, electrical stimuli are now considered as one of the most promising tools for non-chemical methods for the regeneration of neural tissue [140]. One of the major issues is the development of an electroactive biomaterial with excellent biocompatibility, which should support cell growth and contribute to electrical signal transfer. Carbon based materials (e.g., graphene and carbon nanotubes (CNTs)) are expected to play a pivotal role in neural regeneration because of their outstanding physical, chemical, and mechanical properties [141]. Although many studies have tried to improve the biocompatibility of CNTs, they have many safety issues, causing potential oxidative stress, free radical production, peroxidative product accumulation, DNA damage, and inflammation. Such biocompatibility problems are important obstacles to overcome for the potentially wide applications of CNTs in ND therapy [142]. In contrast, because of their electrical features, they represent a promising tool for nerve regeneration.

Graphene consists of a layer of carbon atoms arranged in a hexagonal honeycomb lattice; in particular, graphene-family nanomaterials have been identified as new biomaterials for several biomedical applications such as nanocarriers, biosensors, and the neural regeneration of excitable cells [141]. To improve cell-substrate adhesion, graphene needs to be coated with a precoating layer such as laminin, collagen, Matrigel, and poly-l-lysine. Li and colleagues used graphene coated with poly-l-lysine in a hippocampal culture model and found that the scaffold induced the upregulation of growth-associated protein 43, contributing to neurite outgrowth [143]. Cell viability and morphology were evaluated to exclude possible adverse effects of the graphene. An important factor to be considered is the surface topography and the mechanical properties of the microenvironment: Solanki and colleagues intriguingly reported that a grid pattern promoted neural differentiation, while a square pattern promoted glial differentiation [144]. The topography is important for the guidance of neurite growth. Lee’s group developed a system aimed at obtaining a very precise line pattern with a 15 μm width and 8 μm spacing. Such topography was reported not only to enhance the adhesion and growth of primary hippocampal neurons, but also to influence the direction of neurite outgrowth [145]. The models previously described were composed of a 2D layer of graphene, but this material can be used for the generation of 3D scaffolds. One of the main aims of tissue engineering is to mimic the 3D architectures of tissues and organs, but at the same time, the structure has to ensure efficient cellular metabolism, oxygen and nutrient transportation, and waste removal [146]. Intriguingly, 3D graphene scaffolds were proven to induce much milder neuroinflammation compared to 2D films, allowing for a better biocompatibility [147]. Examples of the 3D graphene-based scaffold are the so-called graphene foams that were demonstrated to support the growth and differentiation of neural cells [148]. Graphene family nanomaterials are usually used as bioactive supporting material, but they can be utilized as interfacing materials for neurons in composite scaffolds. A substrate of graphene-incorporated chitosan showed enhanced adhesion, proliferation, and, more importantly, neurogenesis of adipose-derived hMSCs [149]. Such effects are due to both its nanotopology and electrical properties. Neural regeneration of graphene materials was tested in a small number of in vivo models. Wang and colleagues tested a nanofibrous scaffold combined with graphene in a rat model with a sciatic nerve injury. They found that nerve regeneration and consequent functionality were similar to the gold standard autograft [150]. In the study of Qian and colleagues, the effects of graphene nanoscaffolds in nerve repair were evaluated both in vitro and in vivo, showing excellent functional and morphological recovery, again equivalent to those of autografts. Interestingly, they found a pro-angiogenic characteristic of graphene, but to study the potential mechanism behind this key phenomenon, further studies are needed [151].

CNTs are sheets of graphite rolled into cylindrical tubes with a diameter range of 0.4–2 nm, and with lengths much longer, ranging from hundreds of nanometers to micrometers [152]. They can be divided into single-walled CNTs (SWCNTs) and multiwalled CNTs (MWCNTs), depending on their geometry. As graphene, CNTs can conduct electrical stimuli, making it suitable for neural tissue engineering. CNTs are particularly used as additives in biomaterials to provide conductive properties to electrical inert materials. In some works, researchers applied electrical stimuli to the scaffold in order to improve the effect of CNTs. Imaninezhad and colleagues found that a scaffold composed of polyethylene glycol and MWCNTs improved neurite outgrowth and neurite length, which can be significantly enhanced by electrical stimulation by 2-fold and 1.8-fold [153]. Another similar work confirmed the hypothesis that electrical stimulation improved not only neurite extension, but also proliferation, cellular migration, and intracellular connections, which are all critical for nerve regeneration [154]. CNTs can be used without giving electrical stimulation as they provide the biomaterial with a differential potential that was found to be effective enough to allow neurite outgrowth and neural regeneration. Shesthra and colleagues developed a polyurethane-silk scaffold added with MWCNTs and demonstrated that it significantly enhanced P12 neural differentiation and maturation with axonal regrowth [155]. SWCNTs can provide enough conductivity properties for neural differentiation. Bordoni and colleagues found that SH-SH5Y fully differentiates and generates mature synapses when cultivated on a SWCNTS/cellulose-based scaffold opening for a new approach in neural regeneration [156]. The possibility of providing conductivity without external stimulation is fundamental for tissue engineering because it can easily be provided in vitro, while it is hard to achieve in vivo.

In vivo studies are complicated by the toxicity that can cause CNT-based implantation, although few groups have evaluated the effect of CNTs in an in vivo situation. CNTs were used for both neural and nerve regeneration. For neural regeneration, Marei and colleagues engrafted NSCs isolated from a human olfactory bulb in a rat model. They demonstrated that a co-engraft with CNTs provided support, enhancing their tendency to differentiate into neurons rather than glial cells [157]. For nerve regeneration, two studies demonstrated the positive effects of CNTs. In the first, Ahn et al. used a model of sciatic nerve injury in a rat model and found that in the CNT composite scaffold, the number of regenerated axons crossing the scaffold, the cross-sectional area of the re-innervated muscles, and the electrophysiological findings were all significantly improved [158]. In the second, Lee et al. developed a MWCNT–hydrogel neural scaffold and found that CNTs promoted axon outgrowth [159].

## 3. Concluding Remarks

Neural disorders remain a clinical challenge in the future and biomaterials represent a promising tool for their treatment. As previously stated, biomaterials can be composed of either natural and/or synthetic compounds. The biomaterials used in the studies described above present diverse characteristics, but all of them were reported to be biocompatible, biofunctional, bioinert, biodegradable, and sterilizable. Some of them (i.e., hydrogels and NPs) have been particularly used for regeneration and treatment in the field of NDs because of their specific characteristics. Indeed, hydrogels have been described as optimal scaffolds for the culture and differentiation of stem cells. Moreover, they resulted in being an optimal vector for the delivery of specific growth factors to support cell growth and differentiation. NPs have been reported as promising vectors to deliver many active and protective molecules for the treatment of NDs. In contrast, SAPs, nanofibers, and carbon-based nanomaterials have been reported to be useful in the restoration of nerve injury. Indeed, both SAPs and nanofibers have a fiber-like structure that allows for the correct guidance of axons, promoting and supporting axon outgrowth. Finally, carbon-based nanomaterials, because of their capacity to boost electrical conductivity, have particularly been used for axon restoration.

Although biomaterials were tested in both in vitro and in vivo animal models, many studies have to be conducted before applying such materials for human neural regeneration. Indeed, while biomaterials have many evident beneficial effects, they can also have some issues regarding safety in humans, for example, triggering inflammation or an oxidative stress effect. Great advances are expected to emerge in the near future in the field of biomaterials, allowing for an improvement in the treatments of human neural disorders.

## Supplementary Materials

Supplementary materials can be found at https://www.mdpi.com/1422-0067/21/9/3243/s1.

## Abbreviations

ND	Neurodegenerative Disease
AD	Alzheimer’s Disease
PD	Parkinson’s Disease
ALS	Amyotrophic Lateral Sclerosis
SCI	Spinal Cord Injury
TDP-43	TAR DNA-binding protein 43
BBB	Blood–Brain Barrier
APP	Amyloid Precursor Protein
Aβ	Amyloid Beta
NMDA	*N*-methyl-d-aspartate
SOD1	Superoxide Dismutase 1
FUS	Fused in sarcoma
AAV	Adeno-Associated Viruses
CED	Convection-Enhanced Delivery
MSC	Mesenchymal Stem Cell
UC-MSC	Umbilical Cord Mesenchymal Stem Cell
BMSC	Bone Marrow Stem Cell
ASC	Adipose Stem Cell
ECM	Extracellular Matrix
HA	Hyaluronic Acid
VEGF	Vascular Endothelial Growth Factor
BDNF	Brain-Derived Neurotrophic Factor
GDNF	Glial Cell-Derived Neurotrophic Factor
iPSC	induced Pluripotent Stem Cell
ENSPC	Embryonic Neural Stem/Progenitor Cell
NP	Nanoparticle
PLGA	Poly-(Lactic-co-Glycolic Acid)
NADPH	Nicotinamide Adenine Dinucleotide Phosphate
SAP	Self-Assembling Peptide
CNT	Carbon Nanotube
