# Supplementary material for: Biomaterials in Neurodegenerative Disorders: A Promising Therapeutic Approach
Source: Int J Mol Sci. 2020 May 4;21(9):3243. doi: 10.3390/ijms21093243 (PMC7247337; doi:10.3390/ijms21093243)
Supplement: Supplementary file 1 [file ijms-21-03243-s001.pdf]

Biomaterials in Neurodegenerative Disorders: A Promising Therapeutic Approach

BIOMATERIAL	PATHOLOGY	REFERENCES
Hydrogels	AD	[60], [61], [63], [63]
	PD	[64], [65], [66], [67], [68]
	ALS	[69], [70]
	SCI	[71], [72], [73], [74], [75]
Nanoparticles	AD	[77], [78], [79], [80], [81], [82]
	PD	[83], [84], [85], [86]
	ALS	[87], [88], [89], [90], [91], [92], [93]
	SCI	[94], [95], [96], [97], [98], [99], [100], [101], [102], [103], [104]
SAPs	AD	[109], [110], [111], [112], [113]
	Other NDs	[114], [50]
	SCI	[115], [116], [117], [118], [119], [120], [121], [122], [123]
Nanofibers	NDs	[128], [129], [130], [131], [132], [133], [134], [135], [136], [137], [138]
Carbon-based nanomaterials	Graphene for NDs	[143], [144], [145], [146], [147], [148], [149], [150], [151]
	CNTs for NDs	[153], [154], [155], [156], [157], [158], [159]
